# Surface Treatment Effect on the Mechanical and Thermal Behavior of the Glass Fabric Reinforced Polysulfone

**DOI:** 10.3390/polym16060864

**Published:** 2024-03-21

**Authors:** Galal Sherif, Dilyus I. Chukov, Victor V. Tcherdyntsev, Andrey A. Stepashkin, Mikhail Y. Zadorozhnyy, Yury M. Shulga, Eugene N. Kabachkov

**Affiliations:** 1 Production Engineering and Mechanical Design, Faculty of Engineering, Minia University, El-Minia 61111, Egypt; eng_galal_emad@mu.edu.eg; 2 Laboratory of Functional Polymer Materials, National University of Science and Technology “MISIS”, Leninskii Prosp, 4, Moscow 119049, Moscow Region, Russia; d.chukov@misis.ru (D.I.C.); a.stepashkin@misis.ru (A.A.S.); zadorozhnyy.my@misis.ru (M.Y.Z.); 3 Center for Project Activities, Moscow Polytechnic University, Bolshaya Semenovskaya Str., 2, Moscow 107023, Moscow Region, Russia; 4 Federal Research Center for Problems of Chemical Physics and Medicinal Chemistry, Russian Academy of Sciences, Ac. Semenov Avenue 1, Chernogolovka 142432, Moscow Region, Russia; shulga@icp.ac.ru (Y.M.S.); en.kabachkov@gmail.com (E.N.K.); 5 Osipyan Institute of Solid State Physics, Russian Academy of Sciences, ul. Akademika Osipyana, 2, Chernogolovka 142432, Moscow Region, Russia

**Keywords:** polysulfone, glass fibers, solution impregnation, silanes, mechanical properties, thermal behavior

## Abstract

The chemical structure of the surface of glass fibers, including silanized fibers, was studied. Highly efficient heat-resistant composites were obtained by impregnating silanized glass fiber with a polysulfone solution, and the effect of modification of the surface of glass fibers on the physical, mechanical and thermophysical properties of the composite materials was studied. As a result of the study, it was found that the fiber-to-polymer ratio of 70/30 wt.% showed the best mechanical properties for composites reinforced with pre-heat-treated and silanized glass fibers. It has been established that the chemical treatment of the glass fibers with silanes makes it possible to increase the mechanical properties by 1.5 times compared to composites reinforced with initial fibers. It was found that the use of silane coupling agents made it possible to increase the thermal stability of the composites. Mechanisms that improve the interfacial interaction between the glass fibers and the polymer matrix have been identified. It has been shown that an increase in adhesion occurs both due to the uniform distribution of the polymer on the surface of the glass fibers and due to the improved wettability of the fibers by the polymer. An interpenetrating network was formed in the interfacial region, providing a chemical bond between the functional groups on the surface of the glass fiber and the polymer matrix, which was formed as a result of treating the glass fiber surface with silanes, It has been shown that when treated with aminopropyltriethoxysilane, significant functional unprotonated amino groups NH^+^/NH_2_^+^ are formed on the surface of the fibers; such free amino groups, oriented in the direction from the fiber surface, form strong bonds with the matrix polymer. Based on experimental data, the chemical structure of the polymer/glass fiber interface was identified.

## 1. Introduction

Polysulfone (PSU) is a high-performance engineering thermoplastic exhibiting excellent mechanical behaviors even at elevated temperatures because of the high temperature of glass transition. It finds wide applications because the high resistance to hydrolysis allows it to be used in medicine where autoclave and steam sterilization are required, or in the food industry, in process equipment, and electrical and electronic components because of PSU properties, such as mechanical behavior, flexibility, excellent heat resistance, and often being a prospective alternative to polycarbonate. On the other hand, PSU has low resistance to some solvents and is susceptible to aging which results in rapid softening.

The disadvantages of pure PSU can be eliminated by adding certain reinforcing fillers. For instance, graphene due to its laminar structure and high specific surface area can improve the thermal and mechanical properties of PSU. Ionita et al. [1] first used a phase inversion method to integrate graphene oxide into a PSU matrix. The nanocomposite showed improvement in both thermal and mechanical properties. The same team [2] used ammonia-functionalized graphene oxide (GO-NH_2_) to reinforce PSU, and it was shown that such a filler was uniformly dispersed in the PSU matrix to form exfoliated structures; composites showed improvement in thermal stability, tensile strength and elastic modulus in relation to pure PSU [2].

Pena-Bagamonde et al. [3] analyzed the effect of using reduced graphene oxide (rGO) on the morphological and thermomechanical characteristics of PSU composites produced by solvent-free extrusion. To improve the dispersion of the nanofiller, PSU was prepared into small particles and mixed with rGO. The thermomechanical properties of rGO/PSU composites were improved compared to unmodified rGO/PSU composites; moreover, an increase in the Tg value was observed [3].

The inclusion of nanoparticles as reinforcing elements in polymer matrices is an effective way to regulate the functional characteristics of the material [4]. Khvatov et al. [5] demonstrated the effect of multi-walled carbon nanotubes (MWCNTs) on the properties of PSU-based nanocomposites. The reinforcement of PSU with MWCNT resulted in a slight increase in the tensile modulus and dynamic modulus of MWCNT/PSU at room temperature. However, the presence of MWCNTs in the PSU provided the high-temperature stability of the composite.

The effect of cellulose nanofibers (CNFs) on the microstructure and mechanical properties of epoxy resin and PSU blends was investigated in [6]. Small amounts of CNF increase the toughness and tensile strength due to enhanced interfacial adhesion resulting from hydrogen bonding interactions between CNF and polymer. T_g_ values were significantly increased with the addition of CNF; 0.3 wt.% of CNF increased the T_g_ by 18 °C compared to pure epoxy. Olmos et al. [7] studied the effect of silica nanoparticles on the thermomechanical properties of PSU. It was found that silica nanoparticles had no effect on the T_g_ value, which indicates a weak interaction between the particles and the PSU.

High-performance composite materials often use carbon fibers (CF) as reinforcement due to their exceptional properties. Previously [8] we studied the effect of thermal oxidation of the CF surface on PSU composite behavior. Thermal oxidation was carried out by heating at 300–500 °C for 30 min in an air atmosphere. The shear strength increased by 1.5 times compared to composites with untreated CF. In [9] we reported the effect of the chemical and thermal oxidation of the surface of carbon fibers on the thermal and mechanical properties, as well as the interfacial interaction of PSU/CF composites. CF surface modification leads to a significant increase in thermal and mechanical characteristics compared to composites reinforced with untreated CF [8,9].

In paper [10], we investigated the effect of removing the coating on glass fiber (GF) surfaces using heat treatment on the mechanical and thermomechanical properties of PSU composites. The mechanical properties were investigated using bending and shear tests. To investigate the thermomechanical properties of PSF composites, thermal deflection temperature (HDT) tests and dynamic mechanical analysis (DMA) were carried out. The chemical structure and microstructure of the fracture surface were studied using IR spectroscopy and SEM, respectively. Three fiber contents were used (50, 60 and 70 wt.%). Mechanical and thermomechanical properties have been shown to improve with increasing fiber content. The interfacial interaction in the preheated composites was improved because of coupling agent removal from the GF surface during heat treatment, which improved the properties of the preheated GF-containing composites compared to those reinforced with untreated ones. SEM images showed a good distribution of PSF on the GF surface for the composites with preheated GF.

The mechanical behavior of fiber-reinforced composites depends on the stability of the interface between the matrix and fiber surfaces. Environmental factors, including temperature, humidity and the stress state to which the material is subjected, can result in interfacial adhesion degradation. This is especially important for GF-reinforced materials because these kinds of fibers are highly hygroscopic [11]. Several strategies have been developed to improve the interfacial adhesion between polymers and GF. The first one is to treat the surface of GF using coupling, nucleating or coupling agents, and the second one consists of the introduction of an interfacial compatibilizer into the matrix; additionally, matrix treatment with oxygen plasma can be used [12]. The use of coupling agents that chemically react with both the matrix and the reinforcement elements is the most effective method for chemically bonding the matrix to the encapsulated reinforcement elements. Organofunctional silanes, which can provide the glass surface not only with chemical reactivity but also hydrophobic properties, are the most widely used coupling agents to improve interfacial adhesion in glass-reinforced materials. Their effectiveness depends on the nature and pre-treatment of the substrate, the chemical structure of the silane used, the thickness of the silane layer and the parameter of the glass surface treatment with silane. Silane coupling agents created a strong bond between the surfaces of organic and inorganic materials. The surfaces of the materials used can be modified with silanes to create the desired heterogeneous environments or to combine the volumetric characteristics of different phases into a single compositional structure at the interfaces of such materials [13]. They have been reported to improve interfacial strength and interfacial hydrothermal resistance in composites [14,15]. These two types of functionalities are usually specified in the general formula of the silane coupling agent. The hydrolyzable X-group usually represents an alkoxy, acyloxy, halogen or amino group, which can be hydrolyzed. The hydrolysis produces a reactive silanol group, which can condense on surfaces with other silanol groups, such as on the surface of siliceous fillers, to form siloxane compounds. The organofunctional R-group is a non-hydrolyzable organic radical that can have a significant effect on the functional properties of the surface [16]. The result of the interaction of a substrate with an organosilane may be to change the wetting or adhesive properties of the substrate, use the substrate to catalyze chemical transformations at a heterogeneous interface, order the interfacial region, and change the characteristics of the interface. It is noteworthy that treatment with silanes can also affect the covalent bonding between organic and inorganic substances.

Silanization of the GF surface usually proceeds via GF treatment in the solution of silane; the solvent may be either organic, such as ethanol [17,18,19,20,21,22,23], methanol [24], toluene [25], cyclogexane [26] or tetrohydrofuran [27] or water-based [28,29,30,31]. Silanized GF are widely used for polymer reinforcing. Most of all, epoxy resin is used as a matrix polymer in such composites [17,18,19,20,23,25,29,30]. The use of another matrix polymer, such as polylactide acid (PDA) [24,32,33], polypropylene [22,31], polypropylene oxide [28], polyoxymethylene [22] and polyamide 6.6 [21] were reported also. Filler in such composites may represent GF in a short [20,22,24,27,29,31,32,33], unidirectional [23,25] or fabric [17,18,19,21,28,30] state. Silane treatment of GF allows for an increase in the composite density [18,29], i.e., to decrease the defectivity of the macrostructure. It was observed that silane treatment of GF results in an increase in tensile [18,22,24] and flexural [19,20,30] strength, elastic moduli [18,19,20,24,30] and elongation at the break [22] of composite materials. In a comparative study on the effectiveness of GF in reinforcing polylactide (PLA) using silane coupling agents (GF-S) and graphene oxide (GF-GO) as surface modifiers, Jing et al. [32] found that the use of GF-S significantly increases the mechanical strength and maintains the relatively good toughness of composites. In contrast, GF-GO has demonstrated the ability to act as nucleation centers for PLA crystallites and can slightly increase the elastic modulus of composites. Surface modification affected matrix crystallinity, interfacial adhesion, and fiber length. Wang et al. [33] also examined the mechanical and thermal behavior of silane-modified GF in PLA composites. The results showed that silane-modified GF bonded well to the PLA matrix; increasing the GF content resulted in a slight decrease in the critical length of the GF in the composite. Mechanical tests have shown that GF simultaneously increases the strength, stiffness and toughness of composites.

Thus, literature data show that silane treatment is an effective way to improve the interaction on the boundary between GF and polymer. However, no data on the use of silane-treated GF in PSU-based composites were observed. The present study aimed to use silane treatment of previously reported [10] preheated GF for the further improvement of PSU-based composite properties.

## 2. Materials and Methods

### 2.1. Materials

The matrix material used in this work was polysulfone (PSU) Ultrason S 2010 (BASF, Ludwigshafen, Germany) with a glass transition temperature of 187 °C and a density of 1.24 g/cm^3^. Woven glass fabric (NPO “Stekloplastic,” Moscow, Russia) (T-23 “260 ± 10 g/m^2^, 12 + 1 warp, 8 + 1 weft yarn/cm, 0.27 ± 0.03 mm thickness”) was used as reinforcement. N-methyl-2-pyrrolidone (Eastchem, Changzhou, China), two silane coupling agents (Aminoethylaminopropyltrimethoxysilane (Silane 6020) and Aminopropyltriethoxysilane (Silane 6011)) (Dow Inc., Midland, TX, USA) and acetic acid (JSC “VEKTON”, St.-Petersburg, Russia) were used as reagents.

### 2.2. Silane Treatment of Glass Fibers

For silane treatment, the glass fabric was first preheated in an ambient atmosphere at 350 °C for 1 h to burn off the organic sizing layer applied to its surface, as it was reported in [10]. Silane coupling agents were diluted to 1 wt.% using deionized water. The silanol group is usually unstable in the presence of water, but it is stable in a slightly acidic solution. To conduct this, acetic acid was used to make the solution pH between 3.5 and 5. The silane coupling agent was then hydrolyzed for 30 min using a magnetic stirrer at medium speed, and the glass fiber was immersed in the hydrolyzed silane solution for 30 min. It was then dried at 110 °C for 30 min, followed by 24 h at room temperature.

### 2.3. Composites Formation

**Figure 1:**
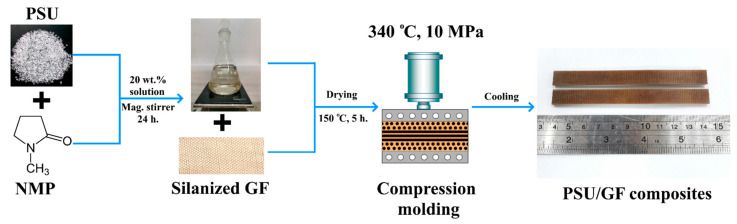
Scheme of preparation of the PSU/GF composites.

A previously reported [10] solution technique was used to obtain composite samples. The scheme of the process is shown in Figure 1. The polymer solution was prepared by dissolving PSU pellets in N-methyl-2-pyrrolidone. The solution was prepared in a 20/80 weight ratio of polymer and solvent for 24 h using a magnetic stirrer. After impregnating the fabrics with the resulting solution, the samples were dried at a temperature of 150 °C for 5 h to remove the solvent before subsequent compression molding at a temperature of 340 °C and a pressure of 10 MPa. Composites were prepared at three-weight fiber-to-polymer ratios (50/50, 60/40, and 70/30 wt.% fiber/polymer).

To ensure the required fiber-to-polymer ratio, the glass fabrics were weighed and based on a known mass, the fabrics were impregnated with the required amount of polymer solution. The mass of the solution (80 wt.% PSU and 3020 wt.% NMP) was calculated so that after removal of the solvent the remaining amount of polymer was 50, 40, or 30 wt.%, which made it possible to obtain samples with fiber-to-polymer ratio of 50/50, 60/40 and 70/30 wt.%.

### 2.4. Investigation Methods

The IR spectra were obtained with a resolution of 4 cm^−1^ and 32 scans were recorded at room temperature in the range of 450–4000 cm^−1^ on a Perkin-Elmer “Spectrum Two” FT-IR spectrometer (Waltham, MA, USA) with an ATR attachment.

XPS spectra were obtained using a Specs PHOIBOS 150 MCD9 electronic spectrometer (Specs, Berlin, Germany), equipped with a magnesium anode (Mg Kα radiation is 1253.6 eV). When recording spectra, the vacuum in the spectrometer chamber did not exceed 3 × 10^−10^ Torr, and the source power was 150 W. The spectra were recorded in the constant transmission energy mode (40 eV for the survey spectra and 10 eV for individual lines). The survey spectra were recorded with a step of 1 eV, and the spectra of individual lines—with a step of 0.05 eV. When calculating the elemental composition, relative sensitivity factors were used.

The microstructure of the fracture surface was studied using a scanning electron microscope (VEGA 3 TESCAN) (TESCANORSAYHOLDING, a.s., Brno-Kohoutovice, Czech Republic) in backscattered electron imaging mode. Before SEM examination, the samples were coated with a thin layer (10–15 nm) of carbon in a sputtering unit to drain the accumulated charge from the surface of the samples.

**Figure 2:**
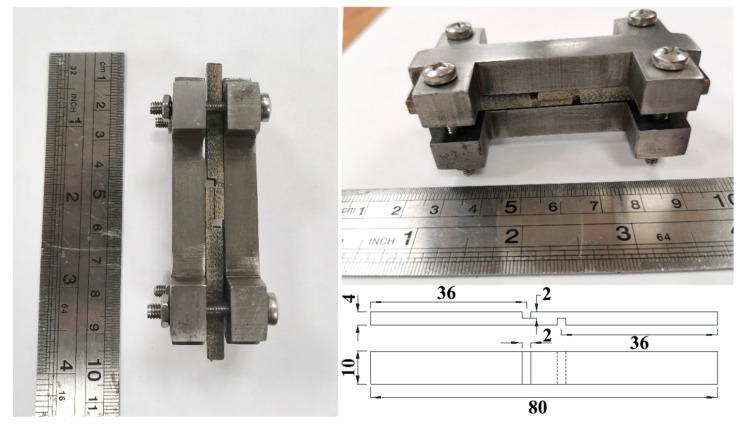
Experimental setup and geometric parameters (in mm) of samples for interlaminar shear tests.

To prepare specimens for interlaminar shear tests (ILSS), the composites were cut using a band saw into specimens measuring 110 × 10 × 4 mm (according to ASTMD 3846 [34]) and 80 mm long, and then 2 mm wide grooves were cut on opposite sides of the specimen using an end mill, 2 mm deep and at a distance of 10 mm from each other. Testing was carried out using custom-made clamps (Figure 2) on a Zwick/Roell Z020 (Zwick GmbH & Co., Ulm, Germany) universal testing machine with a crosshead speed of 1.3 mm/min in compression test mode. The measurements were carried out at room temperature; six samples were tested for each type and composition of composites.

Tensile tests of the specimens were carried out on a Zwick/Roell Z020 universal testing machine using a minimum of six specimens measuring 110 × 10 × 2 mm in accordance with ISO 527:2009 [35] at a crosshead speed of 10 mm/min at room temperature using a 20 kN load cell and MultiXtens (Zwick GmbH & Co., Ulm, Germany) contact strain measurement systems, at least six samples were tested. From the recorded stress-strain curves, tensile strength, elongation at break, and Young’s modulus were calculated.

To study the flexural mechanical properties, such as stress and strain, as well as elastic modulus, the standard ISO 14125:1998 [36] three-point bending method was used. Samples measuring 110 × 10 × 2 mm were cut using a band saw; the span width during testing was 80 mm. The tests were carried out on a Zwick/Roell Z020 universal testing machine at a constant crosshead speed of 10 mm/min. For each type and composition of composites, at least six samples were tested; tests were carried out at room temperature. It should be noted that for different fiber-to-polymer ratios, there was some difference in thickness (up to 0.2 mm between 50/50 and 70/30 composites), but all the obtained data were calculated using the actual cross-sectional area of each sample.

To study the dynamic mechanical properties, a dynamic mechanical analyzer DMAQ800 (TA Instruments, New Castle, DE, USA) was used. For DMA tests, samples 45 × 2 × 2 mm in size were used, and at least 6 samples were tested. Measurements were carried out using a double-converter clamp at a frequency of 1 Hz and a strain of 0.1%, over a temperature range of 30 to 220 °C; the heating rate was 2 °C/min.

To characterize the thermal resistance of the prepared composites, thermal deflection tests were carried out in accordance with ISO 75 [37]. The measurements used specimens 80 × 10 × 4 mm in size at a load of 1.8 MPa and a span width of 64 mm, at least six samples were tested. HDT testing was carried out using the Instron CEAST 6910 HDT/Vicat tester (Norwood, MA, USA). The maximum deflection in the HDT test was set to 1 mm.

## 3. Results and Discussion

### 3.1. Investigation of Glass Fiber Surface

**Figure 3:**
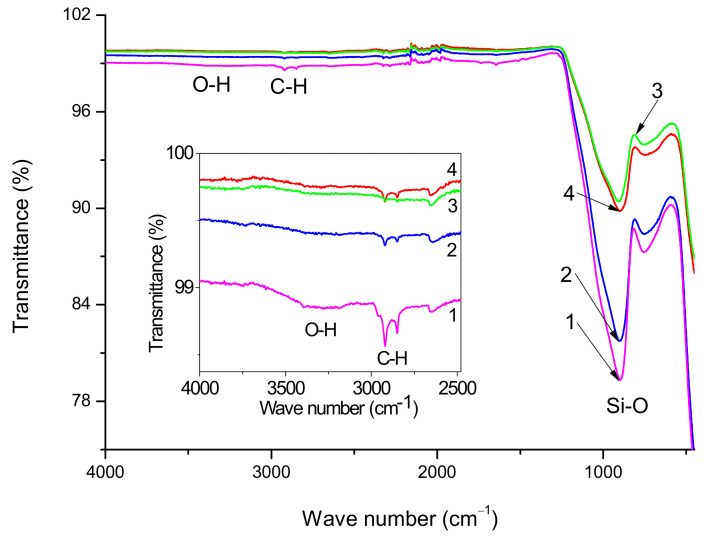
FT-IR spectra of the initial (1), preheated (2) and treated with 6020 (3) and 6011 (4) silanes glass fiber surface; The inset shows a fragment of the spectra in the region of stretching vibrations of O-H and C-H bonds.

Figure 3 shows the FT-IR spectra of initial, preheated and silanized GF. The most intense absorption bands in all spectra are determined mainly by the glass matrix, which to a first approximation, is the same for all samples, and does not depend on the treatments undertaken. Such kinds of spectra with strongly revealed bands related to Si–O bonds in the glass body are typical for GF [38]. Since in the case of the glass fibers under study, the sizing composition has a paraffin base, noticeable peaks are observed in the spectra of the initial GF in the region of 2969–2831 cm^−1^, caused by stretching vibrations of the C–H bonds of the CH_2_- and CH_3_-groups, also the spectra of initial GF contain the OH absorption bands in the region of 3100–3400 cm^−1^. Comparing spectra 1 and 2 (inset in Figure 3), it may be concluded that preheating removes the main part of the sizing because the intensities of absorption bands caused by stretching vibrations of O–H and C–H bonds decrease.

**Figure 4:**
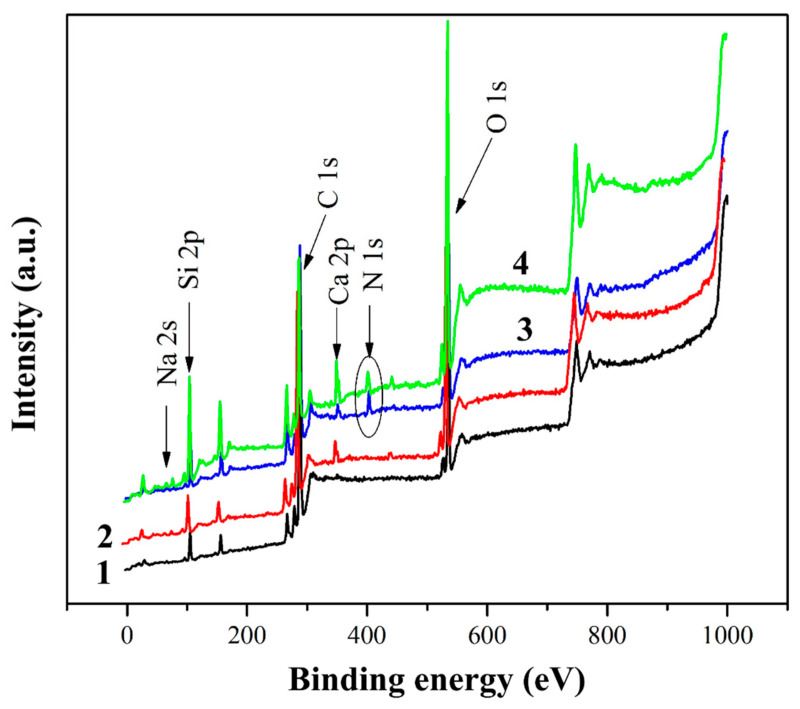
XPS spectra of the initial (1), preheated (2) and treated with 6020 (3) and 6011 (4) silanes glass fiber surface.

For a more detailed study of the effect of treatments on the chemical composition of the GF surface, XPS studies were carried out in the present paper. Figure 4 shows the XPS spectra of the initial, heat-treated and silanized glass fibers. In addition to the expected elements (silicon and oxygen), carbon, sodium and calcium are also observed on the surface of the original glass fiber. One might think that most of the surface carbon is due to the sizing agent that is used in fiber production technology when drawing the glass melt through a die. However, some contribution to the intensity of the C1s peak is also made by uncontrolled hydrocarbon contaminants that form on the surface when storing samples in the air. Calcium and sodium are components of inorganic glass fibers; the presence of these elements in the XPS spectra indicates that the thickness of the sizing agent and contaminations layer is less than 10 nm (XPS probing depth).

From a comparison of spectra 3 and 4, it follows that the hydrocarbon content in the composite treated with silane 6011 is greater than in the composite treated with silane 6020, as judged by the intensity of the C-H absorption bands (2922 and 2848 cm^−1^). IR spectra confirmed the presence of polysiloxane chains on the surface of GF treated with silanes. Thus, stretching vibrations of N-H and C-H bonds appear in the form of absorption bands in the ranges of 3500–3300 cm^−1^ and 2959–2842 cm^−1^, respectively [39]. The peak at 2170–2110 cm^−1^ is associated with the stretching vibration of Si-H bonds [40]. Formation of such bonds can mean that long polysiloxane chains sometimes break away from the “silicon cross” at a Si-O bond rather than an O-C bond. Due to the small amount of silane concentration used, the amine bonds are hardly detectable and further were confirmed by XPS analysis.

**Figure 5:**
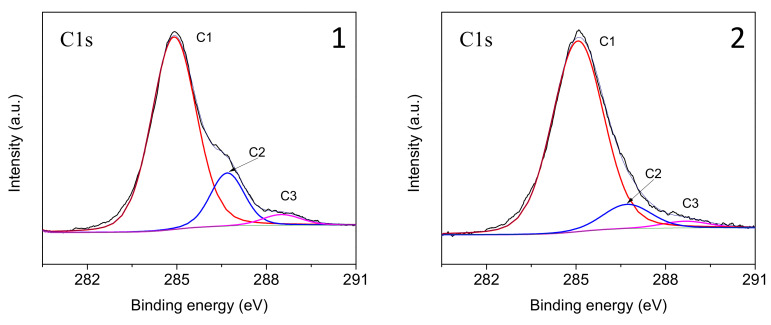
High-resolution C1s X-ray spectra of the initial (**1**) and preheated (**2**) glass fiber surfaces.

Heat treatment results in a noticeable change in the elemental composition of the GF surface. While a fairly high carbon content (73.54 at.%) was found in the initial GF, heat treatment of glass fibers resulted in a decrease in the carbon concentration on the surface to 60.32 at.%. To analyze the C1s region, a high-resolution spectrum was used, shown in Figure 5. The C1s spectrum of the surface of the original fibers mainly corresponds to a paraffin sizing agent, which is typical for CB. After heat treatment, a sharp change in the shape of the C1s spectrum was found, and the intensity of the C–C/C–H peak decreased with a significant increase in the C–O and C–OH/C=O peaks.

In accordance with the literature data [41,42,43,44], the C1s line is described by four peaks caused by carbon atoms that do not have bonds with oxygen (C1), have one bond with the oxygen atom (C2), and have two bonds with the atom/oxygen atoms (C3) and having three bonds with oxygen atoms (C4), respectively. The relative positions of the peaks vary somewhat among different authors, in particular, the average values of binding energies for atoms C1, C2, C3 and C4 are 285, 287, 288 and 289–290 eV, respectively [44]. Let us note that in the XPS spectra of the studied samples, the C4 peak was practically absent (Figure 5). After heat treatment, a sharp change in the shape of the C1s spectrum was detected, which consists of a significant decrease in the relative intensities of peaks C2 and C3 and an increase in the half-width of the main peak C1, which proceeds due to the partial conversion of the sizing into carbon black. In addition, the silicon content on the surface increased, indicating that the sizing coating was largely removed from the surface of the glass fiber during heat treatment, confirming the effectiveness of this method in removing sizing components [45,46].

**Figure 6:**
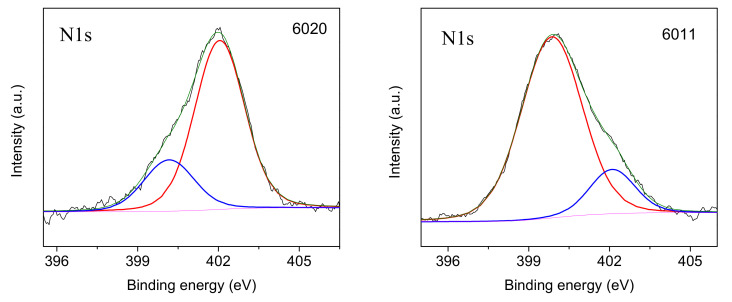
High-resolution N1s X-ray spectra of glass fiber after treatment with silanes 6020 and 6011.

It was expected that the surface chemistry of the glass fibers would change after chemical treatment with silanes. The distinct N1s peak at 400–402 eV for the aminosilane-treated GFs indicates good adhesion of the aminosilane coupling agents to the GF surface. The increase in carbon content in silanized GF was due to CH_2_ bonds, the content of which in silane 6020 is higher than in silane 6011. The increase in Si content is due to the chemical bond between GF and silanes, that is, in the presence of silanes on the GF surface, Si-O-Si bonds are formed. The Si content was lower in fibers silanized with silane 6020 because the longer CH_2_ chains of silane 6020 overlap the Si that is located on the surface of the fibers. The N1s spectra presented in Figure 6 make it possible to identify the features of the chemical bonds formed by silanes 6020 and 6011 on the GF surface. Two peaks of chemical bonding were detected involving protonated amino groups with high binding energy and unprotonated amino groups with low binding energy. The formation of the protonated amino group at 402 eV is due to the reaction between the polar NH/NH_2_ groups present in the silanes and the OH groups present on the surface of the GF, resulting in the formation of NH_2+_/NH_3_^+^ functional groups. The surrounding OH groups limited the mobility of these protonated amines, and hence their ability to react with the polymer matrix. In the case of silane 6020, most of the amine groups were in the protonated state (denoted as N^+^, peak at 402 eV), in this case, there were more of them than unprotonated amine groups (denoted as N, peak at 400.23 eV), which are free amino groups oriented in the direction from the surface of the GF, that is, for this silane, most of the amino groups reacted with the GF. On the contrary, in GF treated with silane 6011, a large amount of unprotonated amine was found, that is, in this case, there are many free amino groups on the GF surface. These free amino groups are able to readily react with the functional groups of the polymer matrix because they are not inhibited by surrounding polar molecules. As will be shown below, this explains the significantly higher positive effect of GF treatment with silane 6011, revealed by studies of the physical, mechanical and thermophysical characteristics of composites than for silane 6020.

### 3.2. ILSS Investigation of Composites

**Figure 7:**
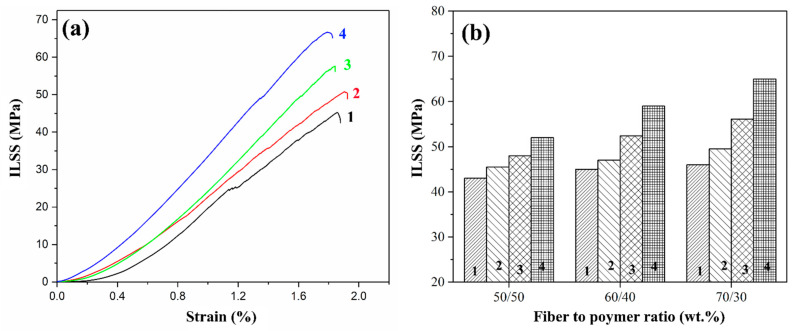
Typical test curves for 70/30 (wt.%) composites (**a**) and ILSS values (**b**) of the PSU-based composites reinforced with initial (1), preheated (2) and treated with 6020 (3) and 6011 (4) silanes GF.

Figure 7 shows typical test curves for 70/30 (wt.%) composites and the ILSS values for composites reinforced with initial, pre-heat-treated, and silanized glass fibers with different fiber-to-polymer ratios. The ILSS values for a composite reinforced with initial GFs are low and almost do not depend on the fiber/polymer ratio in the composite. Also, it should be noted that on the stress-strain curves, step-like displacements can be found which are more noticeable for the initial glass fiber-reinforced composites. The displacements are related to the initiation of the delamination processes during the tests and the minimum values of the corresponding stress (25 MPa) were found for the initial GF-reinforced composites. This behavior is explained by the weak adhesion between the polymer and the GF due to the degradation of the paraffin-based sizing agent during the compression molding process that results in initiating the delamination at relatively low-stress values and the lowest ILSS values among the composites studied. After the size was removed by heat treating the fibers, the composites with pretreated GF showed an increase in shear strength to a value of 49.5 MPa for composite with a 70/30 fiber-to-polymer ratio (wt.%). The improvement in the interfacial interaction during heat treatment can be attributed to the increased ability of the polymer to form strong covalent bonds with the treated GF surface after the removal of the anti-adhesion coating, in addition to the improved physical adhesion of the polymer to the glass fiber.

After GF chemical treatment with silanes, a further increase in ILSS values was observed for the composites. The PSU (70/30) composites showed ILSS of 56.1 and 64 MPa for GF modified with silanes 6020 and 6011, respectively. Thus, the amine groups formed on the GF surface as a result of silanization form an intermolecular hydrogen bond with the polymer matrix, thereby increasing the strength of the interfacial interaction. Based on the results obtained, it can be concluded that silane 6011 has a greater number of accessible open bond sites that can connect to polymer molecules compared to silane 6020.

### 3.3. Tensile Tests of Composites

**Figure 8:**
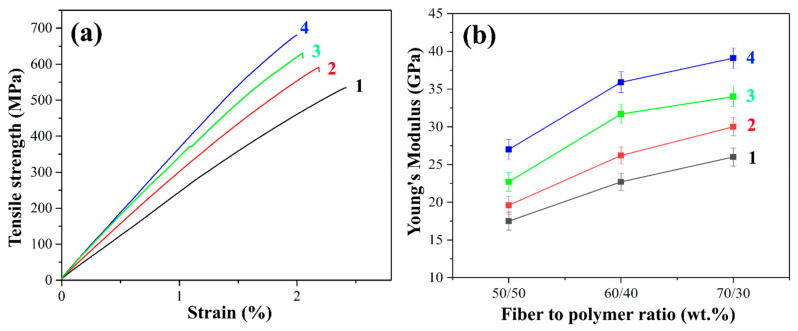
Stress-strain curves for fiber-to-polymer ratio of 70/30 (wt.%) composites (**a**) and Young modulus (**b**) for samples reinforced with initial (1), preheated (2) and treated with 6020 (3) and 6011 (4) silanes GF.

It is well known that in polymer composites the content of the reinforcing filler is one of the main factors affecting the mechanical properties. The fiber content greatly influences the stiffness, strength, and other performance properties of the composite. Higher glass fiber content results in lower relative strain at failure since glass fiber has much higher stiffness and lower ductility. The stress-strain curves and Young’s modulus values of PSU-based composites reinforced with initial and heat-treated GF are shown in Figure 8.

The tensile strength of the initial GFs reinforced composites was relatively low due to the poor adhesion between the polymer and the fiber. The delamination of the matrix from the fiber due to poor adhesion results in the reduced efficiency of load transfer from the matrix to the fiber, which in turn, results in low tensile strength. Heat treatment of GF leads to a significant increase in tensile strength compared to composites reinforced with the initial GFs. This increase ranged from 7 to 13% for PSU-based composites reinforced with heat-treated GF compared to the initial GF. The maximum tensile strength of 590 MPa was achieved in composites with a fiber/polymer ratio of 70/30 wt.%.

In addition, Young’s modulus increased by 11 to 17% for composites reinforced with preheated GF compared to those reinforced with initial GF, the maximum value of 30 GPa was obtained for preheated GF filled 70/30 (wt.%) composites compared to 26 GPa for a composite of the same fiber content reinforced with the initial fibers. This can be explained by the fact that the heat treatment of the GF prevents the matrix from peeling off from the fiber. It should be noted that after heat treatment, the brittle fracture of the composite surfaces was observed. Thus, the tests carried out confirmed the improvement in adhesion at the fiber–polymer interface as a result of removing the paraffin sizing agent by heat treatment. The reason for this increase is the increased interaction between the functional groups located on the surfaces of GF and PSU molecules. The interaction between these functional groups contributes to two mechanisms of adhesion enhancement. The first of these mechanisms is improving the wettability of the fibers due to the removal of the sizing agent, which promotes the more uniform impregnation of the fibers with the matrix polymer. The second mechanism is the improvement of the physical and chemical interaction at the fiber–polymer interface, which ensures efficient load transfer between the matrix and the reinforcing fiber.

Figure 8a shows typical stress-strain curves for composites reinforced with initial, pre-heat-treated and silanized GF fabrics with a filling degree of 70 wt.%. Composites reinforced with silane-treated GF exhibit the highest tensile strength. Thus, the tensile strength for composites reinforced with glass fibers treated with silanes 6020 and 6011 was 628 and 680 MPa, respectively, which is 19 and 28% greater than the strength of composites reinforced with the initial GF (530 MPa). Young’s modulus of the PSF-based composites reinforced with silane 6011-treated fibers is more than 1.5 times higher than that of composites with the initial fibers. The maximum value of Young’s modulus was 39.1 GPa for 70/30 (wt.%) fiber-to-polymer ratio composites reinforced with silane 6011-treated GF, while for the same composites filled with untreated GF, the Young’s modulus was of 26 GPa. The improvement in mechanical properties as a result of silane treatment proceeds due to an increase in the adhesion between components.

As shown above (Section 3.1), reactive nitrile groups are present on the surface of silanized GF, which favors the interfacial interaction between GF and the polymer. The increase in adhesion proceeds via various mechanisms, including increased hydrophilicity, covalent bonding, and interpenetrating networks formed by functional groups, depending on the type of silane used [46,47]. Composites reinforced with silane 601-treated GF exhibited better tensile strength due to the formation of hydrogen and covalent bonds, which provide the highest level of adhesion among the possible mechanisms for improving interfacial interaction when modified with silanes. The data obtained are in good agreement with the ILSS test data (see Section 3.2) for composites reinforced with silanized GF.

### 3.4. Flexural Tests of Composites

**Figure 9:**
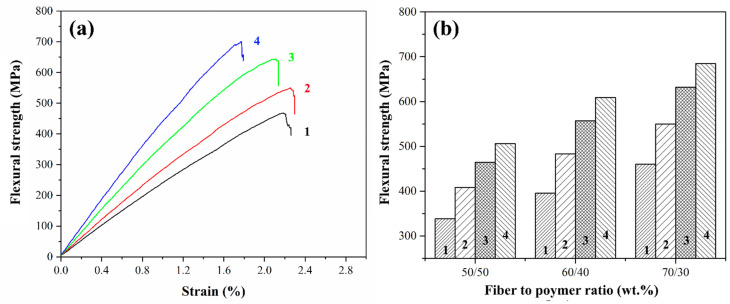
Typical test curves for 70/30 (wt.%) composites (**a**) and flexural strength magnitudes (**b**) for composites reinforced with initial (1), preheated (2) and treated with 6020 (3) and 6011 (4) silanes GF.

Flexural strength is often used to evaluate interfacial interaction in the longitudinal or transverse direction of the fiber. Flexural tests were carried out to evaluate the stiffness and flexural strength of the composites. A comparison of the flexural strength values for composites reinforced with variously treated GF is presented in Figure 9. A trend was observed toward an increase in flexural strength when heat-treated GF was used. This increase for composites reinforced with preheated fibers ranged from 19.5 to 22% compared to composites reinforced with the initial GF. The maximum flexural strength was found to be 550 MPa for fiber to polymer ratio of 70/30 (wt.%) composites. The ILSS tests indicate that the strength of these bonds is higher than that of composites reinforced with initial GF, see Section 3.2. Thus, the use of heat-treated GF improves the interfacial interaction between the polymer and GF due to the removal of the paraffin sizing agent, which is accompanied by an increase in the mechanical properties of the resulting composites.

Composites reinforced with silanized fibers exhibited 37–41% and 49–54% greater flexural strength for composites reinforced with GF treated with silanes 6020 and 6011, respectively, than for composites with initial GF. The improvement in the flexural strength of the composite can be attributed to several mechanisms. The silane coating makes the surface of the glass fiber hydrophilic, which improves wettability and ensures uniform impregnation with the polymer solution. Second, the functional group-driven interpenetrating networks formed between the polymer and silanized GF can enhance the interfacial bond strength. Third, Si-OH formed by hydrolysis of silane sizing agents can interact with -OH on the fiber surface and react with each other to form strong -Si-O-Si- bonds, which enhance the interfacial interaction between the polymer and GF.

It can be concluded that the fiber-to-polymer ratio 70/30 wt.% showed the best mechanical properties for composites reinforced with preheated and GF. It was observed that the heat treatment of GF leads to an improvement in the mechanical properties of composites by 10–20% compared to composites reinforced with initial fibers, while chemical treatment of GF with silanes can increase the mechanical properties by 1.5 times compared to composites reinforced with initial GF.

### 3.5. SEM Investigation of Composite Structure

**Figure 10:**
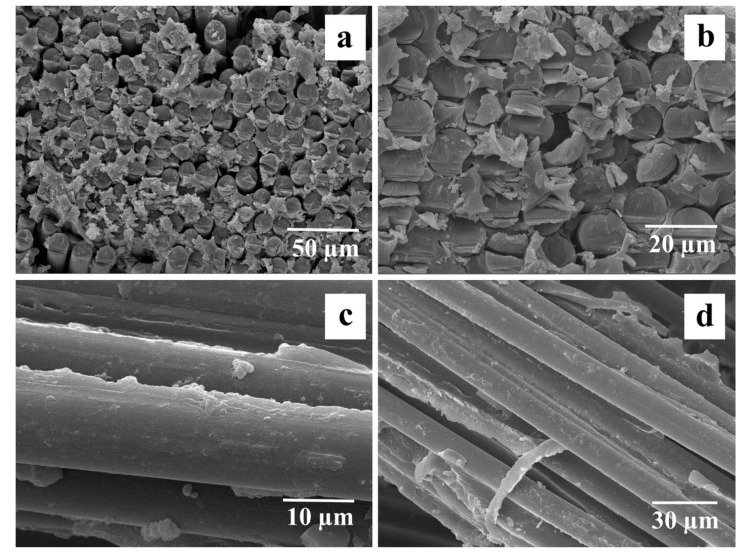
SEM micrographs for 70/30 (wt.%) composites reinforced with (**a**), preheated (**b**) and treated with 6020 (**c**) and 6011 (**d**) silanes GF.

Figure 10 shows SEM images of the fracture surface of GF-reinforced composites. The surface of the initial GF (Figure 10a) was smooth after destruction, which is an indicator of poor interfacial interaction between the matrix polymer and the reinforcing substance (glass cloth). This may be due to the degradation of the sizing agent during the preparation of composites at a temperature of 340–350 °C, which reduced the possibility of covalent bonding between the polymer and the hydroxyl group on the surface of the fibers.

The tensile strength of the composites filled with initial GF was relatively low due to the weak adhesion between the polymer and the fiber (see Section 3.3), as shown in Figure 10a. The delamination of the matrix from the fiber due to poor adhesion results in a reduced efficiency of load transfer from the matrix to the fiber, which in turn, results in low tensile strength. The heat treatment of GF leads to a significant increase in tensile strength compared to composites reinforced with the initial GF. After heat treatment of glass fiber, the Young’s modulus of the composites increased by 11–17%, the maximum values were 30 GPa for heat-treated GF-filled 70/30 (wt.%) composites compared to 26 GPa for composites of the same composition but reinforced with initial fibers. This can be explained by the fact that the heat treatment of the GF prevents the matrix from peeling off from the fiber.

Figure 10b shows that there are polymer particles adhered to the surface of the fibers on the surface of the preheated fibers. Their presence is associated with the formation of polymer bridges between the matrix and fibers, which is an indicator of good adhesion in the resulting composite. This improvement in interfacial adhesion was facilitated by the removal of a coating from the surface of the glass fiber, which can interfere with the interaction between the polymer and the fabric, and its removal increased the expected adhesion sites on the surface of the fibers. As it has been experimentally observed in [48], the optimal heat treatment regime for the studied GF to remove the sizing composition from the point of view of the strength of the resulting composites is heating at 350 °C for 1 h. High adhesion of the matrix to preheated fibers is clearly visible in the micrographs of the fracture surface of the composite shown in Figure 10b, which indicates an improvement in interfacial interaction and a significant increase in the efficiency of uniform load transfer from the matrix to the fiber. It should be noted that after heat treatment, the brittle fracture of the composite surfaces was observed. Thus, the tests carried out confirmed the improvement in adhesion at the fiber–polymer interface as a result of removing the paraffin sizing agent by heat treatment. The reason for this increase is the increased interaction between the functional groups located on the surfaces of GF and PSU molecules. The interaction between these functional groups contributes to two mechanisms of adhesion enhancement. The first of these mechanisms is the improvement of fiber wettability due to the removal of sizing, which promotes more uniform impregnation of the fibers with the matrix polymer, as shown in Figure 10b. The second mechanism is the improvement of the physical and chemical interaction at the fiber–polymer interface, which ensures efficient load transfer between the matrix and the reinforcing fiber.

After chemical treatment, oligomeric siloxane chains were formed on the surface of the fibers (light particles in Figure 10c,d). The formation of such chains acts as additional surface areas in contact with the polymer and creates more bonding bridges. Initially, silane coupling agents interact with the glass surface through hydrogen bonding with the hydroxyl groups of the glass surface. Subsequently, networks of siloxane bonds are formed, creating an interpenetrating network at the surface, which can diffuse into the matrix during composite fabrication.

As shown in Section 3.3, the Young modulus of composites reinforced with silane 6011-treated fibers is more than 1.5 times higher than that of composites with initial fibers. The improvement in mechanical properties as a result of silanization is due to an increase in the level of adhesion between components. As shown in the micrographs (Figure 10c,d), silanized GF has a rougher surface; in addition, as shown above, reactive nitrile groups are present on the surface of silanized GF, which favors the interfacial interaction between GF and the polymer. Composites reinforced with silane 6011-treated 6011 GF exhibited better tensile strength due to the formation of hydrogen and covalent bonds, which provide the highest level of adhesion among the possible mechanisms for improving interfacial interaction when modified with silanes. The data obtained are in good agreement with the ILSS test data (see Section 3.2) for composites reinforced with silanized GF.

### 3.6. DMA Analysis of Composites

**Figure 11:**
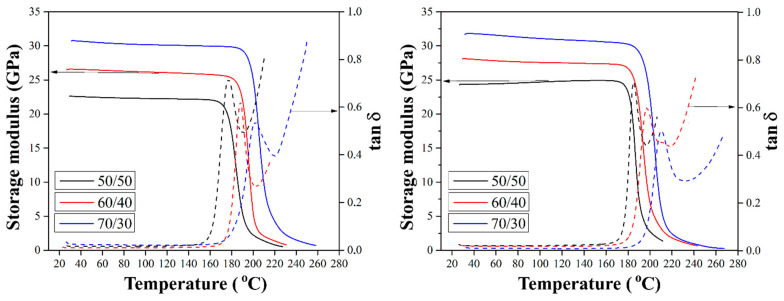
Storage modulus and tan δ temperature dependences for composites with various fiber-to-polymer ratios (wt.%) reinforced with GF treated with silane 6020 (**a**) and silane 6011 (**b**).

To analyze the thermophysical properties of the composites, studies were carried out using the DMA method. Figure 11 shows the elastic modulus (E’) and loss tangent curve of composites reinforced with silanized GF. The elastic modulus characterizes the measure of the elastic response of the composite, and tan δ is equal to the ratio of the loss modulus (E”) to the elastic modulus (E’), being a characteristic of the viscoelastic properties of the material. This method of thermal analysis allows for the measurement of the mechanical properties of the material during its periodic deformation and makes it possible to determine the thermal behavior of the material under cyclic loads.

The elastic modulus values remained on a plateau in the temperature range up to the glass transition temperature (Tg), and in the Tg region, which is the transition zone from the glassy to fully viscous state, a sharp decrease in the modulus value was observed. It was shown that the elastic modulus for all composites increases with increasing fiber-to-polymer ratio, which is associated with an increase in the stiffness and thermal stability of the composites with increasing GF content in the composites. According to the results obtained for composites reinforced with preheated GF, the elastic modulus for composites with fiber to polymer ratio of 70/30 (wt.%) was found to be 26 GPa [10], which is expected to be higher than the elastic modulus for composites with the initial GF. Heat treatment of GF results in an increase in composite thermal stability due to an increase in the rigidity of the composite and improved interfacial interaction. It was also shown that heat treatment of the GF makes it possible to shift the temperature of the tan δ peak towards higher temperatures (by 6–18 °C, compared to composites reinforced with the initial fibers) [10]. This is explained by the fact that reinforcement with preheated fibers reduces the free volume and reduces the mobility of the polymer chains due to the strong interfacial interaction between the GF and the polymer. The increase in interfacial interaction between the fibers and the matrix means that the chain mobility decreases even further, resulting in an increase in the tan δ peak temperature.

The effect of the chemical treatment of CF on the elastic modulus and tan δ of the composites is shown in Figure 11. A noticeable increase in the values of the elastic modulus was found compared to composites reinforced with the initial and preheated GF at the same degrees of filling. As shown in Figure 11, the elastic moduli of the composites with a fiber-to-polymer ratio of 70/30 (wt.%) reinforced with GF silanized with silane 6020 and silane 6011 were 29.7 and 33.5 GPa, respectively, which is expected to be higher than the previously reported [10] elastic modulus of composites containing the initial GF, which was of 22.4 GPa. The increase in the mechanical properties of composites upon silane treatment of GF is due to various mechanisms, including an increase in hydrophilicity, covalent bonding, intermolecular entanglement, and the interaction of secondary bonds, depending on the type of silane binder. For silane 6011 composites, hydrogen bonding and covalent bond formation increase the interfacial strength. This silane has a primary amine structure that forms particularly strong hydrogen bonds with the carboxyl and hydroxyl end groups and reacts with the hydroxyl end groups of PSU. The increased strength of the interfacial bond between silanized hydrocarbons and the polymer, limiting the movement of segments of the molecular chain, increases the elastic modulus and the ability of the material to accumulate energy. It is well known that the tan δ peak amplitude decreases as the interfacial interaction between the filler and matrix improves. Therefore, the peak tan δ value of the same test material can characterize its internal structure and the features of interfacial interaction in the material. The results obtained show that the tan δ maximum decreased with chemical treatment of the GF as a result of improved adhesive interaction.

Moreover, the glass transition temperature (Tg), which was defined as the peak temperature of tan δ, increased for silanized composites compared to composites filled with initial GFs, as shown in Table 1. A decrease in tan δ means that the mobility of the polymer chains decreased due to increasing the bond between fiber and polymer. The improvement achieved through the use of silanized GF increased thermal stability, which increased the Tg of the composites.

### 3.7. HDT Tests of Composites

**Figure 12:**
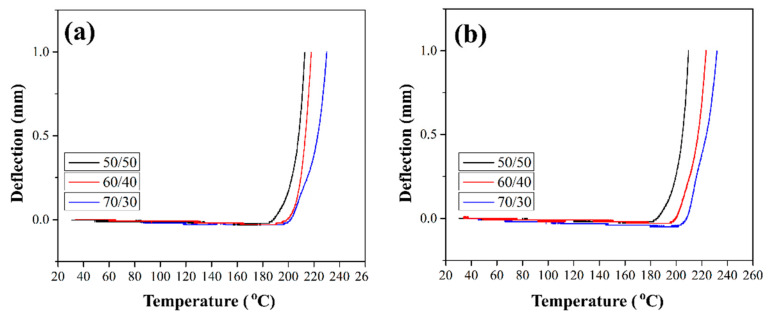
HDT temperature dependencies for composites with various fiber to polymer ratios, reinforced with GF treated with silane 6020 (**a**) and silane 6011 (**b**).

Product design requires knowledge of the thermal deflection temperature (HDT) of materials, which characterizes the thermal stability (absence of significant deformation) of the material when operating under load. HDT tests on composites reinforced with silanized GFs were conducted to study the deformation behavior of the composites under temperature. The maximum deflection was set at 1 mm. Figure 12 shows the HDT curves for composites reinforced with the silane-treated GF. The HDT values for all composites are given in Table 1. It can be seen that the deflection remains close to zero up to temperatures close to Tg (see Section 3.6), while above Tg it sharply increases. In [10] it was shown that HDT for composites reinforced with preheated GF increased by 8–13 °C compared to the HDT values of composites reinforced with the initial GF. Thus, HDT increases as a result of improved stiffness and thermal stability of the material. The deflection value acts as an indicator of thermal stability. Composites reinforced with GF after treatment with silane 6020 showed an increase in HDT by 21–25 °C (Table 1). The best HDT values were obtained for composites reinforced with GF after treatment in silane 6011; the increase in the thermal deflection temperature relative to composites filled with initial GF was 30–35 °C. This improvement is mainly due to the structural properties of the branched chains formed on the surface of GF during the silanization. Branched chains strongly influence the interfacial adhesion between the fiber and the polymer, which leads to an increase in the thermal stability of the composites.

## 4. Summary and Conclusions

**Figure 13:**
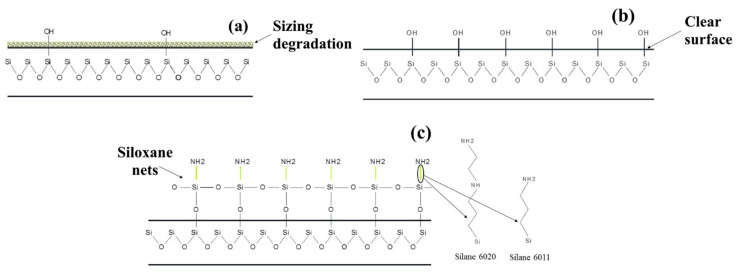
Chemical structure of the surface of the initial (**a**), preheated (**b**) and silanized (**c**) glass fibers.

ILSS is an important indicator of the interfacial interaction between fiber and polymer. Shear tests were conducted on composites reinforced with initial and treated glass fibers, see Section 3.2. The ILSS values for a composite reinforced with initial GFs are low and practically do not depend on the fiber/polymer ratio in the composite. This behavior is attributed to the weak adhesion between the polymer and the GF due to the degradation of the paraffin-based sizing during the compression molding process, as shown in Figure 13a. This is also confirmed by the morphology of the fracture surface of the composite with the initial GF, shown in Figure 10a; it can be seen that when the composite is destroyed, the GF is torn out from the volume of the polymer matrix, which indicates weak adhesion in composites with the initial GF and explains the low values of mechanical properties in tension and bending for composites reinforced with the initial GF. After the sizing was removed by heat treatment of the fibers, the composites showed an increase in shear strength to values of 49.5 MPa for the composites with fiber to polymer ratio of 70/30 (wt.%). The improvement in interfacial interaction during heat treatment can be attributed to the increased ability of the polymer to form strong covalent bonds with the treated GF surface after sizing removal, as shown in Figure 13b, in addition to the improvement in the physical adhesion of the polymer to the glass fiber. The improvement in the adhesion of the matrix to the surface of preheated GF is confirmed by the microphotograph in Figure 10b. After the chemical treatment of GF with silanes, a further increase in ILSS values was observed for the composites. The 70/30 (wt.%) composites showed an ILSS of 56.1 and 64 MPa being reinforced with GF modified with silanes 6020 and 6011, respectively. The increase in ILSS is due to a chemical bond caused by the formation of an intermolecular hydrogen bond between the components, in addition to the physical bond formed due to the interpenetrating network at the phase interface (Figure 13c), which manifests itself in the form of microvolumes of the polymer matrix formed on the GF surface, like in Figure 10c,d. Thus, the amine groups formed on the surface of the carbon fiber as a result of silanization form an intermolecular hydrogen bond with the polymer matrix, thereby increasing the strength of the interfacial interaction. Based on the results obtained, it can be concluded that silane 6011 has a greater number of accessible open bond sites that can connect to polymer molecules compared to silane 6020.

In the present study, we observe that the use of silane coupling agents made it possible to increase the thermal stability of the composites, ensuring the stability of the mechanical properties of the composites up to 200 °C. Mechanisms have been identified that provide improvement in the interfacial interaction between glass fiber and the polymer matrix after thermal and chemical treatment of glass fiber. It has been shown that an increase in adhesion occurs both due to the uniform distribution of the polymer on the surface of the fiber and due to improved wettability of the fibers by the polymer. It was found that as a result of treating the surface of the GF with silanes, an interpenetrating network was formed in the interfacial region, providing a chemical bond between the functional groups on the surface of the GF and the polymer matrix. It has been established that a more effective improvement in the performance properties of the composites in the case of using silane 6011 compared to silane 6020 is associated with long CH_2_ chains in the structure of silane 6020, which limit adhesion, as well as the formation of a large number of free amino groups on the surface of SV treated with silane 6011. It has been shown that after treatment with silane 6011, a significant number of functional unprotonated amino groups NH_2+_/NH_3_ are formed on the GF surface. Such free amino groups, oriented in the direction from the surface of the GF, form strong bonds with the matrix polymer. Based on experimental data, the chemical structure of the polymer/glass fiber interface was identified.

## Figures and Tables

**Table 1:** Tg and HDT values for composites reinforced with initial, preheated and silanized GF.

Fiber to Polymer Ratio, wt.%		GF Treatment			
		Initial	Preheated	Silane 6020	Silane 6011
50/50	T_g_	173	185	196	205
	HDT	168	181	190	199
60/40	T_g_	185	194	207	217
	HDT	177	186	198	210
70/30	T_g_	192	198	211	224
	HDT	180	192	205	215

## Data Availability

The data presented in this study are available on request from the corresponding author in accordance with established practice and University requirements.
